# Extracorporeal cytokine adsorption as therapeutic option for immune effector cell-associated neurotoxicity syndrome

**DOI:** 10.1007/s10072-024-07812-1

**Published:** 2024-10-17

**Authors:** Alix Buhlmann, Emanuel Rom, Giovanna Schweiger, Dominik Schneidawind, Sascha David

**Affiliations:** 1 Institute of Intensive Care Medicine, University Hospital Zurich, Rämistrasse 100, Zurich, 8091 Switzerland; 2 Department of Medical Oncology and Hematology, University Hospital Zurich, Zurich, Switzerland; 3 Institute of Anesthesiology, University Hospital Zurich, Zurich, Switzerland

**Keywords:** Hemoperfusion, Blood Purification, Clearance, Removal

## Abstract

With the rising number of patients receiving chimeric antigen receptor T-cells, the treatment of this therapy’s complications is of growing concern to intensivists and neurologists. We used extracorporeal cytokine adsorption as an add-on therapy in a patient suffering from immune effector cell-associated neurotoxicity syndrome. Interleukin-6 level, which as a readily available parameter is generally used to evaluate course of disease, was rapidly reduced using this method. The patient made a full recovery and is still in hematological remission.

With chimeric antigen receptor-T (CAR-T) cells being the growing therapeutic strategy for a variety of hematological diseases, including refractory and relapsed B-cell malignancies [1], neurologists and intensivists are increasingly confronted with their most common and severe clinical complications. CAR-T cells are genetically altered autologous T-cells engineered to express chimeric antigen receptors against specific tumor-associated antigens. Antigen recognition initiates an immune response, often characterized by supraphysiological release of cytokines, leading to high circulating levels of IL-12, IL-6, TNF-α, IL-1β and IL-15 but low levels of anti-inflammatory cytokines like IL-4 and IL-10 [1]. The two most common clinical complications resulting from this overwhelming response are CAR-T-associated cytokine release syndrome (CRS) and the immune effector cell-associated neurotoxicity syndrome (ICANS) [1–4]. Their incidence varies depending on the specific product, but occur in 20 to 70% of CAR-T patients [5]. CRS can lead to vasoplegia, capillary leakage and organ dysfunction and almost always precedes ICANS, which is characterized by neurological symptoms that can vary from light encephalopathy, up to cerebral edema and death [3]. As of today, Tocilizumab is the treatment of choice against CRS, as it blocks peripheral IL-6 receptors [2]. Since it is unable to penetrate the blood brain barrier (BBB), it does not resolve ICANS and it has been postulated that it might even worsen it, by transiently increasing serum IL-6 levels, which might increase IL-6 in the cerebrospinal fluid (CSF) [4]. So far, steroids are the only effective ICANS treatment although concerns were raised that high-dose glucocorticoids might deplete or eradicate the CAR-T cells, which are unproven at present [6]. Concerning the fact that both pathologies are due to a composite cytokine complex not only IL-6, and that Tocilizumab is ineffective beyond the BBB, we used an extracorporeal cytokine adsorption technology (termed CytoSorb^®^) in a critically ill patient suffering from severe ICANS to reduce the overall cytokine load. The 65-year-old patient presented with progressive shock due to CRS on the fourth day after receiving CD19-specific CAR-T cells (YESCARTA^®^, 0.4–2.0 × 10^8^ cells) for refractory diffuse large B-cell lymphoma (DLBCL). Hence, according to the current state of the art, Tocilizumab was initiated at the recommended dosage of four doses of 8 mg/kg (720 mg), 8 hourly and due to a further hemodynamic decline dexamethasone (10 mg i.v., 6 hourly) was started the following day [3, 6, 7]. Disease course and therapy are shown in Fig. 1. Despite receiving Tocilizumab and dexamethasone within a few hours, the vasopressor requirement increased progressively and the patient started showing signs of encephalopathy (word-finding disorder, disorientation to person and place, fine motor skills disorder without paresis). The neurological decline rapidly progressed with loss of consciousness (GCS 3–6), a tonic-clonic seizure as well as epileptiform activity in EEG correlating with a myoclonus and a massively elevated serum IL-6 level was noted. Therefore, we decided to add an experimental treatment with extracorporeal cytokine adsorption to the already running continuous renal replacement therapy (CRRT) circuit. The patient was off vasopressors within 12 h, the next day’s follow up EEG showed no epileptiform activity and two days later the patient was responsive to speech and extubated on the fifth day. We detected a reduction in IL-6 levels over the course of the hemoperfusion, with an initial IL-6 concentration of 7279 ng/l going down to 735 ng/l. In line with previous reports of potential saturation effects on adsorptive surfaces in the context of very high blood levels, we found a declining IL-6 clearance over time [8].

**Fig. 1:**
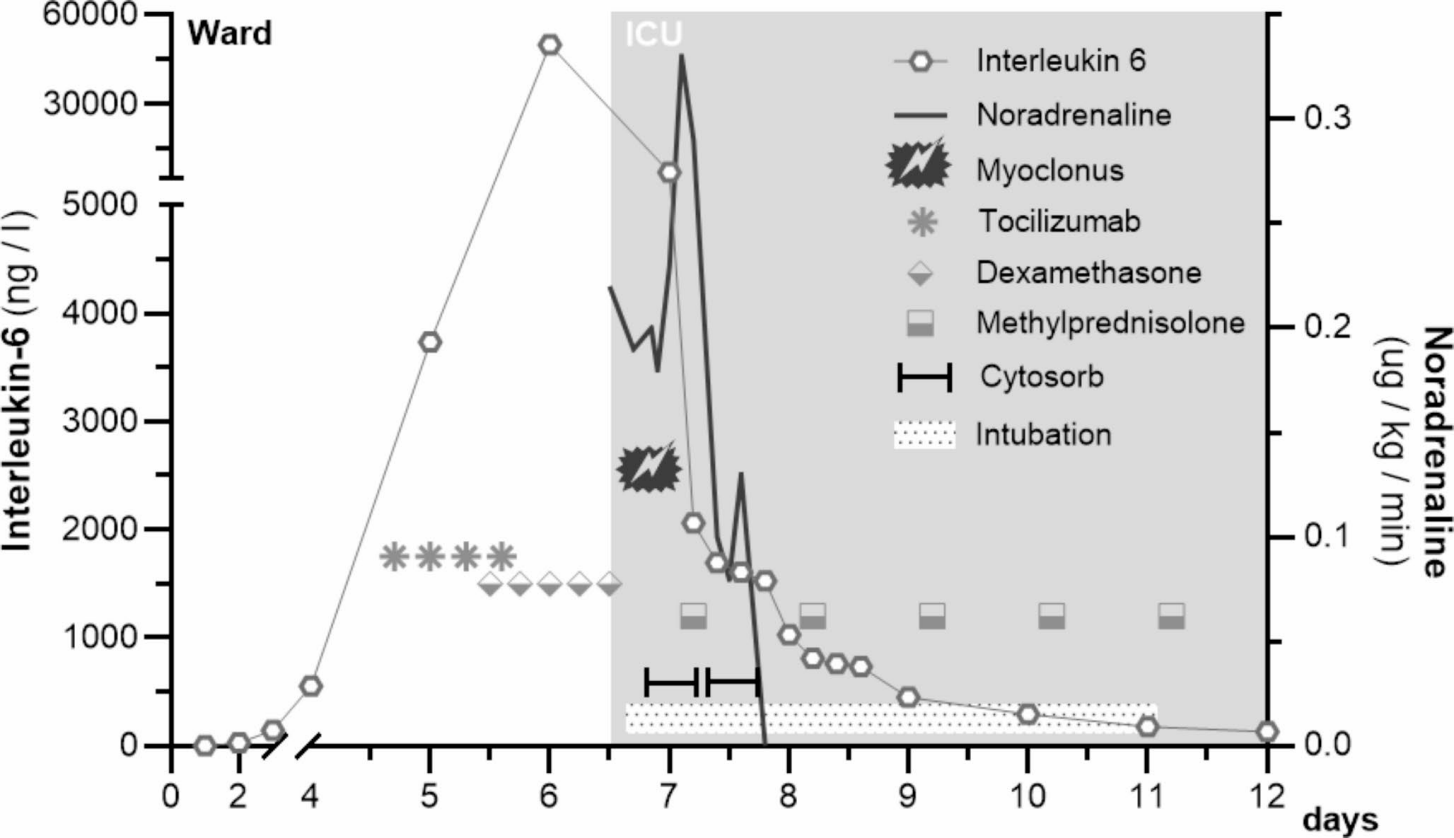
Display of the clinical course and therapies; day 0 is when the patient received CAR-T cells

The rise of IL-6 levels following Tocilizumab administration and its neurotoxic effects have well been described. As Tocilizumab is unable to cross the BBB, it makes pathophysiological sense that patients can present with a worsening neurological state in the course of a CRS treatment. Siltuximab, a monoclonal antibody binding IL-6 and able to penetrate CSF, has been suggested as an alternative therapy, but presently there is not enough data to support this [4]. Furthermore, ICANS therapy with the tyrosine-kinase inhibitor Dasatinib has recently been reported in a single case report [9]. In mouse-models, it induced in CAR-T cells a reversible state of inactivity, which stops cytokine production and counteracted the development of severe CRS. However, the extracorporeal removal of supraphysiological proinflammatory cytokines represents a reasonable approach. We focused on the assessment of IL-6, as it is readily available and often used to evaluate the course of disease, but other pro-inflammatory cytokines taking part on the pathogenesis of ICANS are likewise affected by the chosen unspecific hemoperfusion strategy [10]. The CytoSorb^®^ technology with its polymer beads is size selective up to about 60 kDa and adsorbs primarily hydrophobic substances, which are typical properties of cytokines. Significant removal of albumin, coagulation factors or immunoglobulins was never detected and recently Jansen et al. could not find a negative long-term consequence on immunocompetence [10]. Due to the uncontrolled nature of such a single case description we cannot rule out that clinical improvement might simply reflect the natural course of the disease or a postponed steroid effect and further research needs to be conducted.

Overall, the use of extracorporeal hemoperfusion devices in CRS and ICANS should be further researched as a therapeutic option [11]. A current RCT to test this strategy is recruiting CAR-T patients in Germany (NCT04048434).

## Data Availability

Ethics regulations do not allow data sharing from this article.

## Abbreviations

CAR-T: Cell Chimeric antigen receptor-T cell
IL-6: Interleukine 6
CRS: Cytokine Release syndrome
ICANS: Immune effector cell-associated neurotoxicity syndrome
BBB: Blood brain barrier
CSF: Cerebrospinal fluid
DLBCL: Diffuse large B-cell lymphoma
ICU: Intensive Care Unit
EEG: Electroencephalography
CRRT: Continuous renal replacement therapy
